# Fresh Osteochondral Allograft in a Complex Hoffa Fracture – Case Report

**DOI:** 10.1055/s-0042-1742599

**Published:** 2022-03-07

**Authors:** Thiago Vivacqua, Tito Rocha, José Leonardo Rocha de Faria, Rafael Prinz, Alan Mozella, João Antonio Matheus Guimarães

**Affiliations:** 1 Fowler Kennedy Sports Medicine Clinic, Western University, Ontário, Canadá; 2 Instituto Nacional de Traumatologia e Ortopedia Jamil Haddad, Rio de Janeiro, Rio de Janeiro, Brasil

**Keywords:** allografts, femoral fractures, knee dislocation, osteochondritis

## Abstract

Hoffa fracture (HF) typically involves the posterior femoral condyle in the sagittal plane, with an estimated incidence of 0.65% among femoral fractures. It usually occurs at the lateral femoral condyle in high-energy trauma with axial load force over the distal third of the femur and the knee positioned in more than 90° of flexion. The case reported involved a patient with a complex medial HF and associated knee dislocation after a high-energy trauma. At two years of follow-up radiologic analysis showed a complete bone healing and allograft integration. Transplantation of osteochondral allografts should be considered in cases of complex HF, and it aims at restoring the anatomy of the joint surface to prevent early post-traumatic osteoarthrosis in young patients.

## Introduction

Hoffa fracture (HF) involves the posterior femoral condyle in the sagittal plane, and it has an estimated incidence of 0.65% among femoral bone fractures. 1 It typically occurs after axial load force over the distal third of the femur with the knee positioned in more the 90° of flexion. The lateral femoral condyle (LFC) is often affected in high-energy trauma, and previous valgus limb alignment might be associated with HF at the LFC. 2 However, it has also been reported at the medial femoral condyle (MFC). 1

Anatomic reduction and stable fixation must be prioritized during the surgical approach to HF in order to achieve early joint mobility and decrease the rate of post-traumatic arthritis. Non-anatomical reduction and additional chondrocyte apoptosis are associated with high rate of articular degenerative changes in young patients. The technique of transplantation of osteochondral allografts (OCAs) represents a reasonable surgical approach to repair the anatomy of the chondral surface and restore associated bone loss. 3 Recently, OCAs have been gaining popularity, and have been used in post-traumatic osteoarthrosis (OA), complex fractures, and malunion around the knee joint. 3 The present case report aims to describe an OCA transplant selected as a surgical approach in a complex HF during a motor-vehicle high-energy trauma.

## Case Report

A 27-year-old patient was first admitted to a secondary regional trauma center after being involved in a motor-vehicle accident. The case reported was previously approved by the Institutional Review Board (CAAE: 46596721.2.0000.5273). The radiologic assessment confirmed a right calcaneus fracture, left femoral head fracture, and ipsilateral medial knee dislocation. The clinical examination confirmed a grade-III medial collateral injury, grade-III posterior cruciate ligament lesion, and complete anterior cruciate ligament (ACL) tear, which led to the clinical diagnosis of knee dislocation. The physical examination showed a regular lower-limb pulse and intact nerve function.

**Fig. 1:**
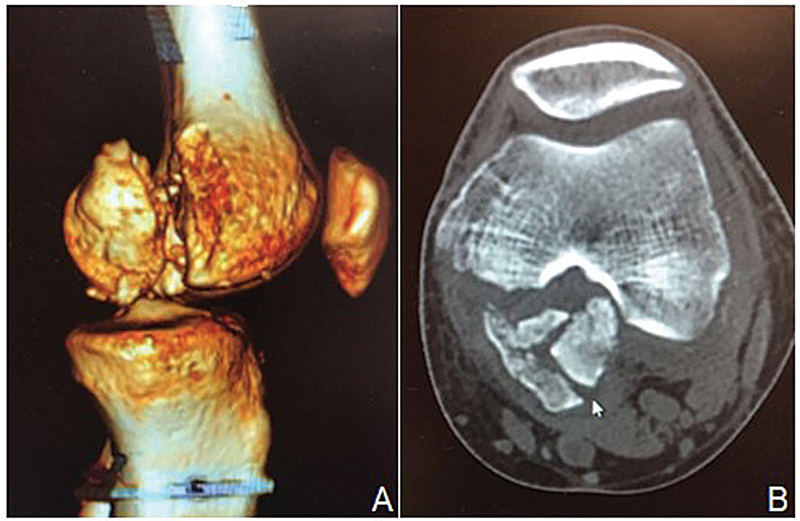
( **A** ) Three-dimensional reconstruction of a preoperative sagittal computed tomography scan. ( **B** ) Preoperative axial computed tomography scan.

A computed tomography (CT) scan of the right knee revealed a complex medial HF – grade 33 B3 according to the classification of the Arbeitsgemeinschaft für Osteosynthesefragen/Orthopedic Trauma Association (AO/OTA) ( Fig. 1 A-B ). Issues such as non-anatomical reduction, post-traumatic OA, and traumatic chondrocyte apoptosis led the senior surgeon to choose allograft tissue transplantation. At the time of the treatment, our group 4 had previously tested and approved an osteochondral-preservation protocol.

**Fig. 2:**
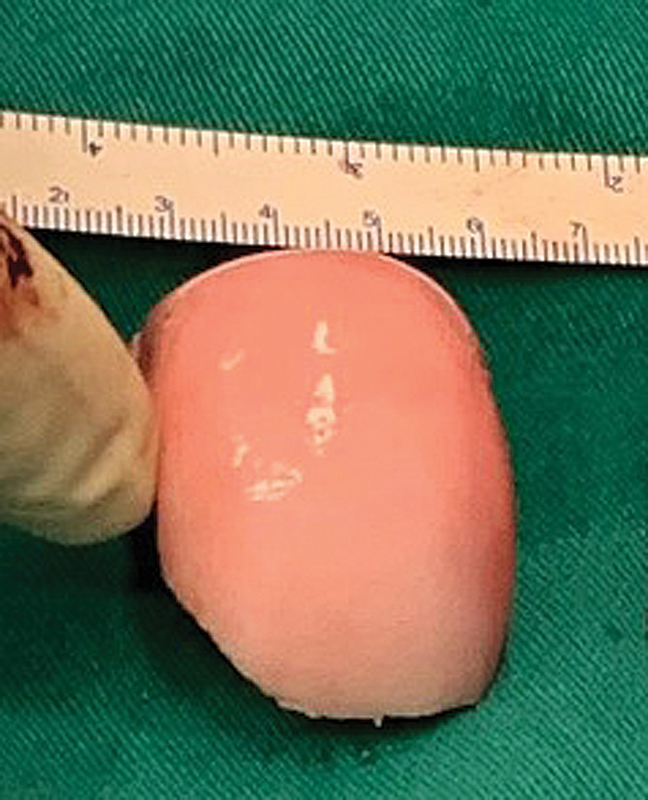
Fresh osteochondral allograft from the posterior femoral condyle.

A left-knee posteromedial approach was selected for the surgical treatment. At the moment of the transplantation, a posterior allogenic femoral condyle was prepared by free-hand technique ( Fig. 2 ).

**Fig. 3:**
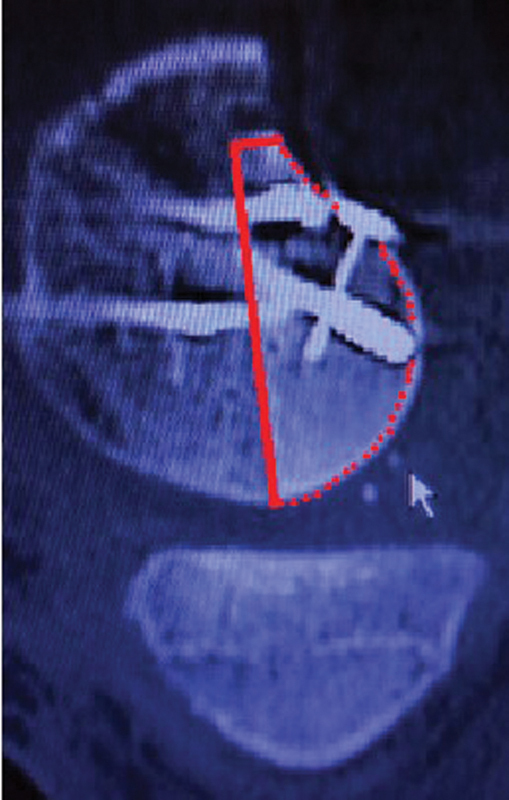
Anatomical reduction confirmed through a postoperative computed tomography scan. The white arrow indicates the osteochondral allograft.

Immediately before the fixation of the transplant, a pulse lavage technique was used to remove cadaveric bone marrow elements to decrease the risk of local allogenic reaction. The Locking Compression Plate (LCP, DePuy Synthes, West Chester, PA, United States) was used for allograft fixation, as well as three small-fragment cortical screws for rotational control. A postoperative CT scan confirmed the anatomical position of the allograft and articular congruence ( Fig. 3 ).

**Fig. 4:**
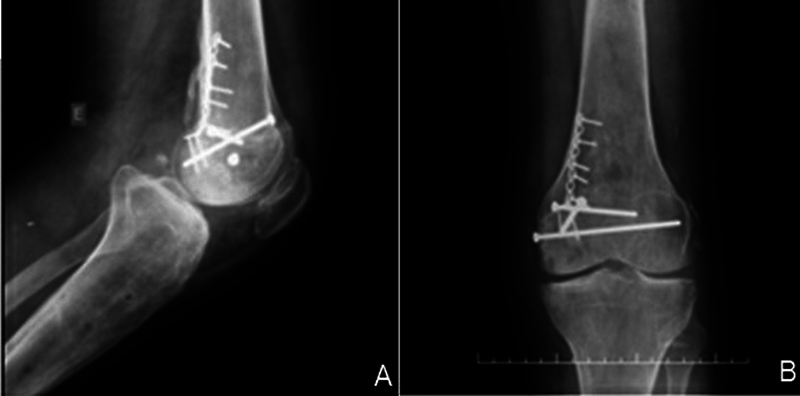
( **A** ) Postoperative lateral view. ( **B** ) Postoperative anterior view.

The postoperative protocol involved six weeks with no weight-bearing and no restriction regarding the range of motion. After six weeks, partial weight-bearing was allowed until twelve weeks of follow-up, with the radiological confirmation of complete bone healing. At two years of follow-up, radiologic assessment confirmed complete allograft integration and anatomical reduction ( Fig. 4 A-B ). No inflammatory reaction or local allogenic rejection was reported during the follow-up. The patient is now on a waitlist to complete the ligament-reconstruction approach regarding his complex knee injury.

## Discussion

Hoffa fractures are typically related to motor-vehicle accidents and falls, depending on the angle of the knee at the moment of the trauma. 1 The patient described was involved in a high-energy trauma accident and suffered multiple fractures. Complete bone healing of femoral head, calcaneus, and posterior condyle fractures were confirmed at the last follow-up.

No surgical approach to HF is often associated with loss of joint mobility, yearly post-traumatic OA and limb malalignment. In the case of simple fractures, anterior-to-posterior fixation using the lateral or medial parapatellar approach represents a reasonable option. 1 Complex medial HF is usually addressed with a medial subvastus approach, while the Swashbucker approach or Gerdy tubercle osteotomy are suggested for complex HF at the LFC. 5 In the case reported, a medial subvastus interval was used, and the medial gastrocnemius was partially released from the MFC.

Unusual associations have been reported regarding HF and injury to the knee extensor mechanism, tibial-plateau fracture, tibial-spine avulsion, patella dislocation, and femoral-shaft fracture. 6 An additional CT scan is recommended for patients with suspected HF, while a magnetic resonance imaging (MRI) scan can be helpful to identify leasion to ligaments or the menisci. 5

The case reported in this manuscript was associated with a complex knee ligament injury. At the time of the present report, we could not complete the reconstruction of the knee ligament to obtain a final functional result. Our group understands that, in the case of OCA transplantation, complete bone integration and healing should be achieved before the arthroscopic reconstruction of the knee. A similar case involving medial HF and multiple ligament injuries was previously reported after a motorcycle accident. 7 Other authors 8 have reported cases of HF and associated lesion to the attachment of the tibial meniscus, cruciate ligaments, and lateral collateral ligament avulsion. Letenneur et al. 9 identified the relationship between HF at the LFC and ACL tear or lateral collateral ligament injury. The association between HF and complex knee injury represents a challenging scenario, witch requires a comprehensive preoperative clinical and radiological diagnosis.

Transplantation of a large OCA has been considered as a reasonable approach after primary cartilage repair failure, post-traumatic OA, and failed fixation of previous articular fractures. 10 Concerns regarding the case reported involves the possibility of incomplete graft integration or chondral delamination, leading to transplant failure. However, it is also known that non-anatomical reduction and traumatic chondrocyte apoptosis result in secondary limb malalignment and early post-traumatic OA. To the best of our knowledge, the presented case is the first report of OCA transplantation after a complex HF.

Transplantation of the OCA resulted in complete bone healing and graft integration in the case reported. Restoration of the anatomical surface of the joint using fresh OCA should be considered in cases of complex HF to prevent early post-traumatic OA in young patients.

## References

[1] Arastu M H Kokke M C Duffy P J Korley R E Buckley R E Coronal plane partial articular fractures of the distal femoral condyle: current concepts in management Bone Joint J 2013 95-B 09 1165 1171 23997126 10.1302/0301-620X.95B9.30656

[2] Gavaskar A S Tummala N C Krishnamurthy M Operative management of Hoffa fractures–a prospective review of 18 patients Injury 2011 42 12 1495 1498 21993368 10.1016/j.injury.2011.09.005

[3] Zouzias I C Bugbee W D Osteochondral Allograft Transplantation in the Knee Sports Med Arthrosc Rev 2016 24 02 79 84 27135291 10.1097/JSA.0000000000000109

[4] Vivacqua T A Prinz R D Cavanellas N Barretto J M de Sousa E B Aguiar D P Protocol for Harvest, Transport and Storage of Human Osteochondral Tissue Rev Bras Ortop (Sao Paulo) 2020 55 02 163 169 32346191 10.1055/s-0039-3400522 PMC7186072

[5] Bagaria V Sharma G Waghchoure C A proposed radiological classification system of Hoffa's fracture based on fracture configuration and consequent optimal treatment strategy along with the review of literature SICOT J 2019 5 05 18 31180317 10.1051/sicotj/2019016 PMC6557153

[6] Kondreddi V Yalamanchili R K Ravi Kiran K Bicondylar Hoffa's fracture with patellar dislocation - a rare case J Clin Orthop Trauma 2014 5 01 38 41 25983467 10.1016/j.jcot.2014.02.001 PMC4009468

[7] Liu Q Wang W Fan W Zhu W Hoffa fracture associated with tibial shaft fracture and multiple ligament avulsion fractures: A case report. Trauma Case Rep 2020 26 100277 10.1016/j.tcr.2020.100277 PMC6970162 31989015

[8] Huang G Zhang M Zhang Y Wang X Zhang M Liu G Hoffa fracture combined with rotational dislocation of the knee joint: A novel case report Medicine (Baltimore) 2021 100 14 e25253 33832086 10.1097/MD.0000000000025253 PMC8036118

[9] Letenneur J Labour P E Rogez J M Lignon J Bainvel J V Fractures de Hoffa a propos de 20 observations. [Hoffa's fractures. Report of 20 cases (author's transl)] Ann Chir 1978 32(3-4):213 219 697301

[10] Sherman S L Garrity J Bauer K Cook J Stannard J Bugbee W Fresh osteochondral allograft transplantation for the knee: current concepts J Am Acad Orthop Surg 2014 22 02 121 133 24486758 10.5435/JAAOS-22-02-121

