# Supplementary material for: Fresh Osteochondral Allograft in a Complex Hoffa Fracture – Case Report
Source: Rev Bras Ortop (Sao Paulo). 2022 Mar 7;59(Suppl 2):e264–8. doi: 10.1055/s-0042-1742599 (PMC11679608; doi:10.1055/s-0042-1742599)
Supplement: Related texts

# Research article: Aloenxerto osteocondral fresco em uma fratura complexa de Hoffa – Relato de caso

**Keywords:** aloenxertos, fraturas do fêmur, luxação do joelho, osteocondrite

## Abstract

A fratura de Hoffa (FH) é típica do côndilo femoral posterior no plano sagital, com incidência estimada de 0,65% nas fraturas do fêmur. De modo geral, a FH é observada no côndilo femoral lateral em traumatismos de alta energia, com força de carga axial sobre o terço distal do fêmur e o joelho em mais de 90° de flexão. O caso aqui relatado ocorreu em um paciente com FH medial complexa e luxação de joelho após um traumatismo de alta energia. Em dois anos de acompanhamento, a análise radiológica revelou consolidação óssea completa e integração do aloenxerto. O transplante de aloenxerto osteocondral deve ser considerado em casos de FH complexa, e visa restaurar a anatomia superfície articular para evitar o desenvolvimento de osteoartrose pós-traumática precoce em pacientes jovens.

## Introdução

A fratura de Hoffa (FH) é típica do côndilo femoral posterior no plano sagital, com incidência estimada de 0,65% nas fraturas do fêmur. 1 Normalmente, é causada por uma força de carga axial sobre o terço distal do fêmur, com o joelho em mais de 90° de flexão. O côndilo femoral lateral (CFL) é frequentemente afetado em traumatismos de alta energia, e o alinhamento prévio do membro em valgo pode estar associado à FH no CFL. 2 No entanto, a FH também é relatada no côndilo femoral medial (CFM). 1

A redução anatômica e a fixação estável devem ser prioridades durante a abordagem cirúrgica da FH, para permitir a mobilidade articular precoce e diminuir a taxa de artrite pós-traumática. A redução não anatômica e a adicional apoptose de condrócitos estão associadas às taxas altas de alterações degenerativas articulares em pacientes jovens. A técnica de transplante de aloenxerto osteocondral (AOC) é uma boa abordagem cirúrgica para o reparo da anatomia da superfície condral e o restauro da perda óssea associada. 3 Nos últimos anos, o AOC ganhou popularidade, e tem sido usado na osteoartrose (OA) pós-traumática, em fraturas complexas, e em casos de má união na região do joelho. 3 Este relato de caso tem como objetivo descrever um transplante de AOC escolhido como abordagem cirúrgica em um caso de FH complexa por traumatismo automobilístico de alta energia.

## Relato de Caso

Um paciente de 27 anos foi primeiramente atendido em um centro regional secundário de traumatismo após sofrer um acidente automobilístico. O caso relatado foi previamente aprovado pelo Comitê de Revisão Institucional (CAAE: 46596721.2.0000.5273). A avaliação radiológica confirmou a presença de uma fratura no calcâneo direito, fratura na cabeça do fêmur esquerdo, e luxação medial do joelho ipsilateral. O exame clínico revelou lesão colateral medial de grau III, lesão do ligamento cruzado posterior de grau III, e ruptura completa do ligamento cruzado anterior (LCA), o que levou ao diagnóstico clínico de luxação do joelho. O exame físico também revelou pulso regular nos membros inferiores e função nervosa intacta.

Uma tomografia computadorizada (TC) do joelho direito mostrou uma FH medial complexa, de grau 33 B3 segundo a Arbeitsgemeinschaft für Osteosynthesefragen */* Orthopedic Trauma Association (AO/OTA) ( Fig. 1 A-B ). Questões como redução não anatômica, OA pós-traumática, e apoptose traumática de condrócitos fizeram com que o cirurgião sênior escolhesse o aloenxerto de tecido. No momento do tratamento do paciente, nosso grupo 4 já havia testado e aprovado um protocolo de preservação osteocondral.

**Fig. 1:** ( **A** ) Reconstrução tridimensional de tomografia computadorizada sagital realizada no período pré-operatório. ( **B** ) Tomografia computadorizada axial pré-operatória.

A abordagem posteromedial do joelho esquerdo foi escolhida para o tratamento cirúrgico. Durante o transplante, um côndilo femoral alogênico posterior foi preparado à mão livre ( Fig. 2 ).

**Fig. 2:** Aloenxerto osteocondral fresco do côndilo femoral posterior.

Imediatamente antes da fixação do transplante, a técnica de lavagem por pulso foi utilizada para remoção do elementos da medula óssea cadavérica para diminuição do risco de reação alogênica local. A fixação do aloenxerto foi feita com placa de compressão bloqueada (Locking Compression Plate, LCP, DePuy Synthes, West Chester, PA, Estados Unidos) e três parafusos corticais de pequenos fragmentos para controle rotacional. Uma TC pós-operatória confirmou a posição anatômica do aloenxerto e a congruência articular ( Fig. 3 ).

**Fig. 3:** Confirmação da redução anatômica na tomografia computadorizada pós-operatória. A seta branca indica o aloenxerto osteocondral.

O protocolo pós-operatório consistiu em seis semanas sem carga e sem restrição quanto à amplitude de movimento. A descarga parcial de peso foi permitida depois de seis semanas até o acompanhamento de doze semanas, quando a consolidação radiológica completa do osso foi confirmada. Em dois anos de acompanhamento, a avaliação radiológica confirmou a integração completa do aloenxerto e a redução anatômica ( Fig. 4 A-B ). Reações inflamatórias ou rejeição alogênica local não foram relatadas neste tempo de acompanhamento. O paciente agora está em lista de espera para concluir a abordagem de reconstrução do ligamento devido à lesão complexa do joelho.

**Fig. 4:** ( **A** ) Vista lateral pós-operatória. ( **B** ) Vista anterior pós-operatória.

## Discussão

A FH está tipicamente relacionada a acidentes automobilísticos e quedas, dependendo do ângulo do joelho no momento do traumatismo. 1 O paciente descrito sofreu um acidente traumático de alta energia e múltiplas fraturas. A consolidação óssea completa das fraturas da cabeça do fêmur, calcâneo e côndilo posterior foram confirmada no último acompanhamento.

A não abordagem cirúrgica para FH está frequentemente relacionada a perda da mobilidade, OA precoce pós-traumática e desalinhamento do membro. Ou desalinhamento secundário dos membros. Nas fraturas simples, a fixação anterior a posterior com abordagem parapatelar lateral ou medial é uma opção razoável. 1 A FH medial complexa é geralmente tratada com abordagem subvasta medial, ao passo que a abordagem de Swashbucker ou a osteotomia do tubérculo de Gerdy são sugeridas para FH complexa no CFL. 5 No caso relatado, o intervalo subvasto medial foi usado, e o gastrocnêmio medial foi parcialmente liberado do CFM.

A FH já foi associada, de forma incomum, a lesão do mecanismo extensor do joelho, fratura do platô tibial, avulsão da coluna tibial, luxação da patela, e fratura da diáfise do fêmur. 6 Uma TC é recomendada em pacientes com suspeita de FH, ao passo que a ressonância magnética pode auxiliar na identificação de lesões em ligamentos ou meniscos. 5

O caso relatado neste manuscrito estava associado a uma lesão complexa do ligamento do joelho. No momento deste relato, não podíamos concluir a reconstrução do ligamento do joelho para obtenção do desfecho funcional. Nosso grupo entende que, no caso do transplante de AOC, a integração óssea completa e a cicatrização devem ser alcançadas antes da reconstrução artroscópica do joelho. Um caso semelhante com FH medial e múltiplas lesões ligamentares foi relatado após um acidente com motocicleta. 7 Outros autores 8 relataram FH e lesões na inserção do menisco tibial, nos ligamentos cruzados, e avulsão do ligamento colateral lateral. Letenneur et al. 9 identificaram a relação entre a FH no CFL e a rotura do LCA ou lesão do ligamento colateral lateral. A associação entre FH e lesões complexas no joelho representam um cenário desafiador, a qual requer uma avaliação pré-operatória abrangente.

O transplante AOC estrutural tem sido considerado como abordagem plausivel após falha em reparo primário da cartilagem. OA pós-traumática, e falha na fixação de fratura articular anterior. 10 Neste relato de caso, as preocupações foram a possibilidade de integração incompleta do enxerto ou deslaminação condral que levasse ao insucesso do transplante. No entanto, sabe-se também que a redução não anatômica e apoptose traumática dos condrócitos provoca o desalinhamento secundário do membro e OA pós-traumática precoce. Pelo que sabemos, este é o primeiro relato de caso de transplante com AOC após FH complexa.

No caso aqui relatado, o transplante de AOC resultou em consolidação óssea completa e integração do enxerto. A restauração da superfície anatômica da articulação utilizando AOCa fresco deve ser considerado na FH complexa para evitar a OA pós-traumática precoce em pacientes jovens.
